# Rethinking rate-related myocardial injury in sepsis: atrial fibrillation, heart rate, cardiac troponin T and long-term mortality

**DOI:** 10.1136/openhrt-2026-004073

**Published:** 2026-06-22

**Authors:** Samantha Lörstad, Louise Essehorn, Johan Engdahl, Jonas Persson

**Affiliations:** 1 Division of Internal Medicine, Karolinska Institutet, Department of Clinical Sciences, Danderyd Hospital, Stockholm, Sweden; 2 Department of Cardiovascular Medicine, Karolinska Institutet, Department of Clinical Sciences, Danderyd Hospital, Stockholm, Sweden

**Keywords:** Atrial Fibrillation, Biomarkers, Inflammation, Epidemiology

## Abstract

**Background:**

Atrial fibrillation (AF), elevated heart rate and elevated high-sensitivity cardiac troponin T (hs-cTnT) are common in sepsis, but their independent and combined prognostic significance remains uncertain.

**Methods:**

This secondary analysis of the retrospective Sepsis and Elevated Troponin cohort study included adults with sepsis requiring vasopressor support admitted to critical care units at Danderyd University Hospital, Stockholm, Sweden, between March 2012 and September 2021. Eligible patients fulfilled Sepsis-3 criteria and had hs-cTnT measured within 48 hours of sepsis onset. Patients were classified as having no AF, pre-existing AF or new-onset AF. AF status and highest recorded heart rate during the first 72 hours were examined in relation to troponin concentrations and 1-year mortality using linear regression, Kaplan-Meier analysis and multivariable Cox proportional hazards regression.

**Results:**

Among 586 included patients, 177 (30%) had pre-existing AF and 112 (19%) developed new-onset AF. Elevated hs-cTnT (≥15 ng/L) was present in 546 patients (93%). Patients with AF had higher hs-cTnT concentrations and higher recorded heart rates. AF accounted for only 1.2% of the variability in hs-cTnT concentrations and 2.2% of the variability in highest recorded heart rate. Heart rate was not associated with hs-cTnT concentrations or mortality. One-year mortality was higher in patients with pre-existing AF (HR 2.4, 95% CI 1.8–3.2) and new-onset AF (HR 1.8, 95% CI 1.3–2.5) than in those without AF. One-year mortality increased progressively across hs-cTnT quartiles, with an additive increase in risk among patients with concomitant AF. After multivariable adjustment, AF and hs-cTnT concentrations remained independently associated with 1-year mortality, whereas highest recorded heart rate did not.

**Conclusions:**

In adults with sepsis requiring vasopressor support, AF and elevated hs-cTnT were associated with increased 1-year mortality, with additive prognostic value independent of highest recorded heart rate. Concomitant AF and troponin elevation identify a high-risk subgroup of sepsis patients whose myocardial injury should not be attributed solely to rate-related demand ischaemia.

**WHAT IS ALREADY KNOWN ON THIS TOPIC**

- Atrial fibrillation and cardiac troponin elevation are common in sepsis and have individually been associated with adverse outcomes. Troponin elevation is often clinically attributed to rate-related demand ischaemia, but the inter-relationships between heart rate, atrial fibrillation and troponin and their combined prognostic significance remain uncertain.

**WHAT THIS STUDY ADDS**

- In adults with sepsis requiring vasopressor support, atrial fibrillation and elevated high-sensitivity cardiac troponin T were independently and additively associated with long-term mortality, whereas heart rate was not associated with troponin concentrations or mortality.

**HOW THIS STUDY MIGHT AFFECT RESEARCH, PRACTICE OR POLICY**

- Concomitant atrial fibrillation and troponin elevation identify a high-risk subgroup of sepsis patients whose myocardial injury should not be attributed solely to rate-related demand ischaemia. Closer cardiovascular risk stratification and structured post-discharge follow-up should be considered in these patients.

## Introduction

 Atrial fibrillation (AF), elevated heart rate and elevated plasma cardiac troponin are common manifestations of sepsis-related circulatory stress, yet their clinical significance remains unclear.^1^,^3^ In practice, troponin elevations during sepsis are often attributed to rate-related demand ischaemia associated with higher heart rates or AF, and structured cardiac follow-up after discharge is not routinely performed.^4^,^6^ However, no guidelines specifically address management of sepsis-associated myocardial injury, leaving uncertainty for critical care physicians and cardiologists regarding short-term and long-term care.^6^

AF and elevated heart rate are recognised markers of illness severity, but whether they directly contribute to myocardial injury or simply co-occur with troponin elevation remains uncertain.^7 8^ Furthermore, the extent to which plasma high-sensitivity cardiac troponin T (hs-cTnT), AF and heart rate independently or jointly relate to long-term mortality is unknown. To address this gap, we investigated the associations of hs-cTnT, AF and highest recorded heart rate, with 1-year mortality in a cohort of critically ill patients with sepsis.

## Methods

### Study population and trial design

The Sepsis and Elevated Troponin (SET) study was a previously reported single-centre retrospective cohort study evaluating the association between plasma hs-cTnT concentrations and mortality in patients with sepsis or septic shock.^3^ Details of patient inclusion are provided in the supplemental material (online supplemental figure S1). The cohort comprised 586 adults with sepsis requiring vasopressor support in the intermediate or intensive care units at Danderyd University Hospital, Stockholm, Sweden, between March 2012 and September 2021. The study was approved by the Swedish Ethical Review Authority.

Patients were identified retrospectively through the electronic health record (EHR). Eligible participants required vasopressor support, fulfilled Sepsis-3 criteria for sepsis or septic shock and had at least one hs-cTnT measurement within 48 hours of sepsis onset.^2 3^ According to Sepsis-3, septic shock was defined as requiring vasopressor therapy to maintain a mean arterial pressure ≥65 mm Hg and serum lactate >2 mmol/L despite adequate fluid resuscitation.^2^

### Data collection and outcomes

Baseline demographic and clinical characteristics, laboratory and microbiological data, in-hospital treatments and mortality were extracted from the EHR. Frailty was assessed using the Clinical Frailty Scale, comorbidity burden using the Charlson Comorbidity Index and disease severity using the Sequential Organ Failure Assessment (SOFA) score. Sepsis onset was defined as the time at which sepsis-related abnormal vital signs were first documented in the EHR.^2^

Electrocardiograms obtained within 8 hours of sepsis onset were classified as normal if sinus rhythm was present with QRS duration ≤0.12 s and no ST-segment or T-wave abnormalities. Patients were classified as having no AF, pre-existing AF (defined by a previously registered International Classification of Diseases [ICD]-10 diagnosis code for AF in the EHR) or new-onset AF (NOAF). AF, whether previously diagnosed or new-onset, was confirmed by ECG or continuous telemetry monitoring within 72 hours of sepsis onset.

Plasma hs-cTnT was analysed at the Karolinska University Laboratory (Stockholm, Sweden) using an electrochemiluminescence assay (Elecsys Assay 2, Roche Diagnostics). Myocardial injury was defined as hs-cTnT above the 99th percentile upper reference limit (≥15 ng/L).^9^ Because AF and heart rate evolve dynamically during sepsis, the highest available hs-cTnT value within 48 hours of sepsis onset was used to reflect cumulative myocardial injury. hs-cTnT concentrations were categorised into quartiles: Q1, 15–41 ng/L; Q2, 42–78 ng/L; Q3, 79–184 ng/L; Q4, >184 ng/L.

The highest recorded heart rate within the first 72 hours of sepsis onset was extracted from the EHR using values documented by nursing staff from continuous telemetry monitoring. Heart rate was analysed as a continuous variable in regression models and dichotomised at 120 beats per minute for Kaplan-Meier analyses. This prespecified threshold was based on physiological data demonstrating rising hs-cTnT concentrations above 110–120 beats per minute in patients with AF, aligned with guideline-recommended rate-control targets (<100–110 beats per min), and was consistent with a previous study in septic and cardiogenic shock that used the same cut-off.^8 10 11^

The primary analysis examined the association between the highest plasma hs-cTnT and all-cause 1-year mortality in patients with and without AF, adjusting for the highest recorded heart rate. Secondary analyses assessed the contributions of AF and heart rate to variability in hs-cTnT.

### Statistical analysis

Baseline characteristics were summarised according to AF category (no AF, pre-existing AF or NOAF). Continuous variables were reported as mean (SD) for normally distributed data and median (IQR) for non-normally distributed data. Categorical variables were presented as counts and percentages. Between-group comparisons were performed using one-way analysis of variance for normally distributed continuous variables and the Kruskal-Wallis test for non-normally distributed continuous variables. Categorical variables were compared using the χ² test.

Time-to-event for 1-year all-cause mortality was estimated using Kaplan-Meier methods, with group comparisons performed using the log-rank test. For stratified Kaplan-Meier analyses, pre-existing AF and NOAF were combined into a single binary AF variable to facilitate visual presentation.

Univariable and multivariable linear regression analyses were used to assess continuous associations, and natural cubic splines were used to evaluate non-linear relationships. Multivariable Cox proportional hazards regression evaluated associations with 1-year mortality. A multivariable regression model was constructed to evaluate the associations between AF, heart rate and myocardial injury. Covariates were selected a priori based on clinical relevance and established associations with sepsis mortality.^3 11 12^ Right-skewed predictors, including hs-cTnT, were natural log-transformed before regression analyses.

Missing data for covariates were negligible. An exception was left ventricular ejection fraction (LVEF), available in 305 patients (52%) who underwent transthoracic echocardiography during hospitalisation. To retain all patients in multivariable analyses, an ordinal variable was created to capture both echocardiography availability and systolic dysfunction: 0, not performed; 1, preserved LVEF (≥50%); 2, mildly reduced LVEF (40–49%); 3, moderately or severely reduced LVEF (<40%).

All tests were two-sided, with p value <0.05 considered statistically significant. Analyses were conducted using IBM SPSS Statistics, V.30.0.0.0 (IBM Corp) and RStudio, V.2024.04.2+764.

## Results

### Baseline characteristics and hospital course

The SET study included 586 patients with sepsis or septic shock requiring vasopressor treatment (online supplemental figure S1).

Patients with AF were older, more frequently male and had greater frailty and cardiovascular comorbidity than those without AF (table 1). Pre-existing AF was associated with a higher burden of chronic cardiovascular disease and lower habitual renal function.

**Table 1:** Baseline characteristics and hospital course

Characteristic	No AF (n=297)	Pre-existing AF (n=177)	New-onset AF (n=112)	P value
Demographics				
Age, years, median (IQR)	68 (58–76)	79 (72–85)	74 (67–80)	<0.001
Female sex, n (%)	125 (42)	52 (29)	39 (35)	0.019
Current or former smoker, n (%)	163 (55)	98 (55)	60 (54)	0.955
BMI, kg/m^2^, median (IQR)	25 (22–29)	25 (22–29)	26 (22–30)	0.582
Clinical Frailty Scale, median (IQR)	4 (3–5)	5 (4–6)	4 (3–6)	<0.001
Medical history				
Previous myocardial infarction, n (%)	29 (10)	40 (23)	15 (13)	<0.001
History of heart failure, n (%)	34 (11)	97 (55)	21 (19)	<0.001
Hypertension, n (%)	150 (51)	135 (76)	69 (62)	<0.001
Diabetes mellitus, n (%)	81 (27)	57 (32)	31 (28)	0.495
Habitual eGFR (mL/min/1.73 m^2^), mean±SD	65±26	53±22	61±22	<0.001
Cancer (previous/current), n (%)	85 (29)	59 (33)	30 (27)	0.419
Charlson Comorbidity Index, median (IQR)	4 (2–6)	6 (5–8)	5 (3–6)	<0.001
Hospital course*				
SOFA score, mean±SD	10±3	10±3	11±3	0.002
Length of stay (days), median (IQR)	4 (2–7)	3 (2–6)	3 (2–7)	0.379
Additional vasopressor, n (%)†	70 (24)	27 (15)	20 (18)	0.075
Highest recorded heart rate (beats per minute), mean±SD	105±21	108±29	115±25	0.001
hs-cTnT (ng/L), median (IQR)	61 (30–144)	86 (47–191)	88 (42–248)	<0.001
NT-proBNP (ng/L), median (IQR) (n=503)	4610 (1549–14500)	14 600 (5802–29200)	10 800 (2712–21675)	<0.001
Lactate, mmol/L, median (IQR)	4.4 (2.5–6.7)	4.6 (2.8–7.3)	5.2 (3.0–8.5)	0.227
CRP, mg/L, mean±SD	276±126	242±117	307±127	<0.001
Creatinine, µmol/L, median (IQR)	186 (117–287)	185 (137–285)	229 (141–331)	0.213
Haemoglobin, g/L, mean±SD	105±19	105±19	109±20	0.139
WBC count, ×10^9^ /L, median (IQR)	18.2 (12–26.2)	18.5 (13.7–25.8)	18.8 (12.4–24.5)	0.573
Met criteria for septic shock, n (%)	255 (86)	161 (91)	107 (96)	0.013
Positive blood culture, n (%)	173 (58)	106 (60)	75 (67)	0.271
LVEF category, n (%)				0.011
Echocardiography not performed	147 (50)	93 (53)	41 (37)	
≥50%	95 (32)	41 (23)	40 (36)	
40–49%	27 (9)	12 (7)	11 (10)	
<40%	28 (9)	31 (18)	20 (18)	

* All clinical, laboratory and SOFA score measurements represent the highest recorded values within the first 48 hours from sepsis onset, except for haemoglobin, for which the lowest recorded value within 48 hours is reported.

† Additional vasopressors were dobutamine, epinephrine, vasopressin, phenylephrine and isoprenaline.

AF, atrial fibrillation; BMI, body mass index; CRP, C reactive protein; eGFR, estimated glomerular filtration rate; hs-cTnT, high-sensitivity cardiac troponin T; LVEF, left ventricular ejection fraction; NT-proBNP, N-terminal pro-B type natriuretic peptide; SOFA, Sequential Organ Failure Assessment; WBC, white blood cell count.

Patients with NOAF demonstrated higher SOFA scores and greater inflammatory burden during sepsis, reflected by higher C reactive protein concentrations. Patients with AF also had higher recorded heart rates, hs-cTnT concentrations and N-terminal pro–B-type natriuretic peptide concentrations. Elevated hs-cTnT concentrations were observed in 546 patients (93%), with values ranging from 4 ng/L to 21 900 ng/L. The range of highest recorded heart rates was 60–174 beats per minute in the no AF group, 44–190 beats per minute in the pre-existing AF group and 62–190 beats per minute in the NOAF group.

### Contribution of AF, heart rate and left ventricular function to variation in hs-cTnT levels

**Figure 1:**
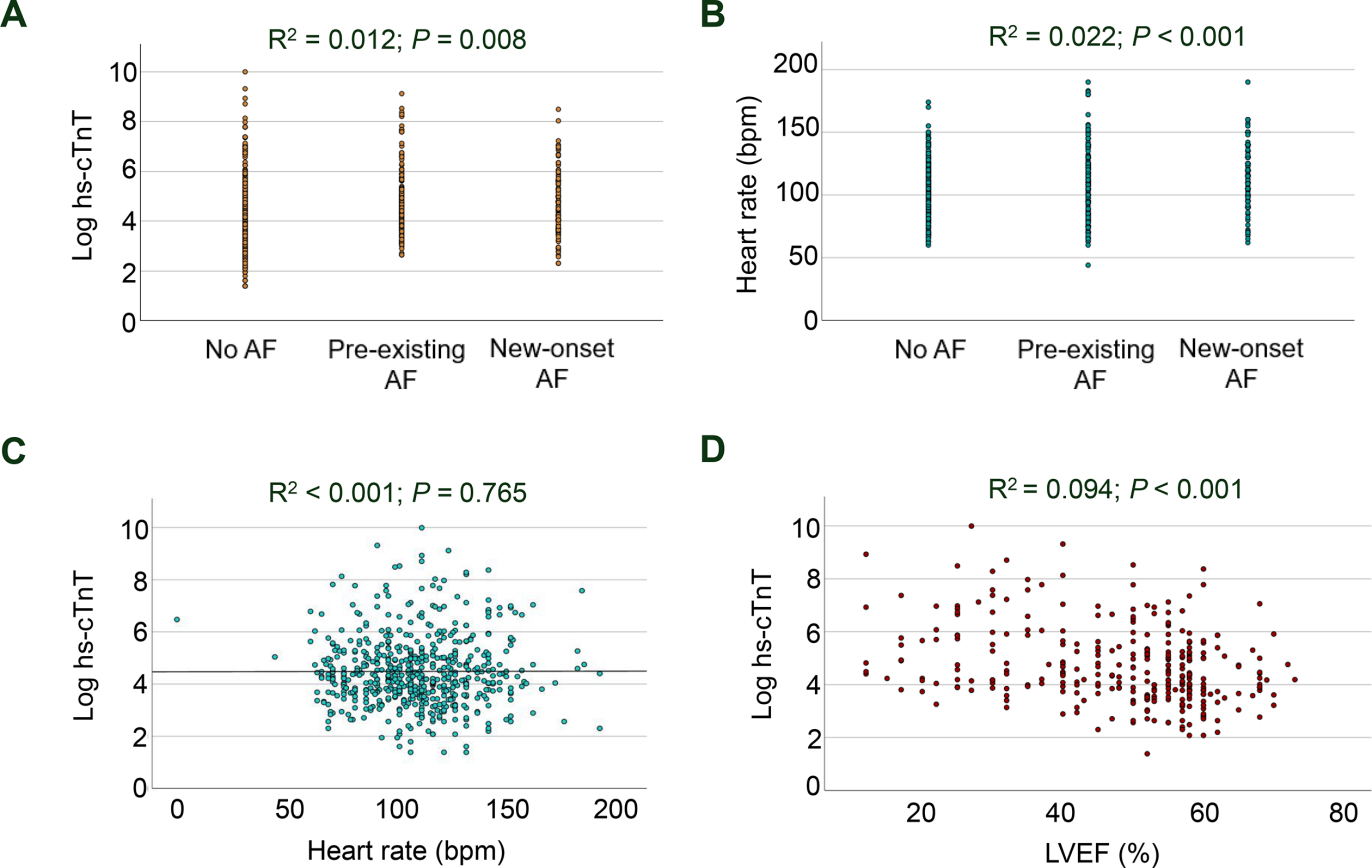
Associations among atrial fibrillation (AF), highest recorded heart rate, left ventricular ejection fraction (LVEF) and log-transformed high-sensitivity cardiac troponin T (hs-cTnT) concentrations. (**A**) Log-transformed hs-cTnT concentrations according to AF group (no AF, pre-existing AF and new-onset AF). (**B**) Highest recorded heart rate according to AF group. (**C**) Relationship between highest recorded heart rate and log-transformed hs-cTnT concentrations. (**D**) Relationship between continuous LVEF and log-transformed hs-cTnT concentrations among patients with echocardiographic data (n=305).

AF status was associated with log-transformed hs-cTnT concentrations, but the effect size was small, explaining only 1.2% of the variability in hs-cTnT levels (figure 1A). Likewise, AF status explained only 2.2% of the variability in highest recorded heart rate (figure 1B). Heart rate itself was not associated with log-transformed hs-cTnT concentrations and accounted for virtually none of the observed variability (R^2^ < 0.001; figure 1C). Among patients with echocardiographic data, lower LVEF was associated with higher log-transformed hs-cTnT concentrations and explained 9.4% of the variability in hs-cTnT levels (figure 1D).

### 1-year mortality by AF, heart rate and troponin

**Figure 2:**
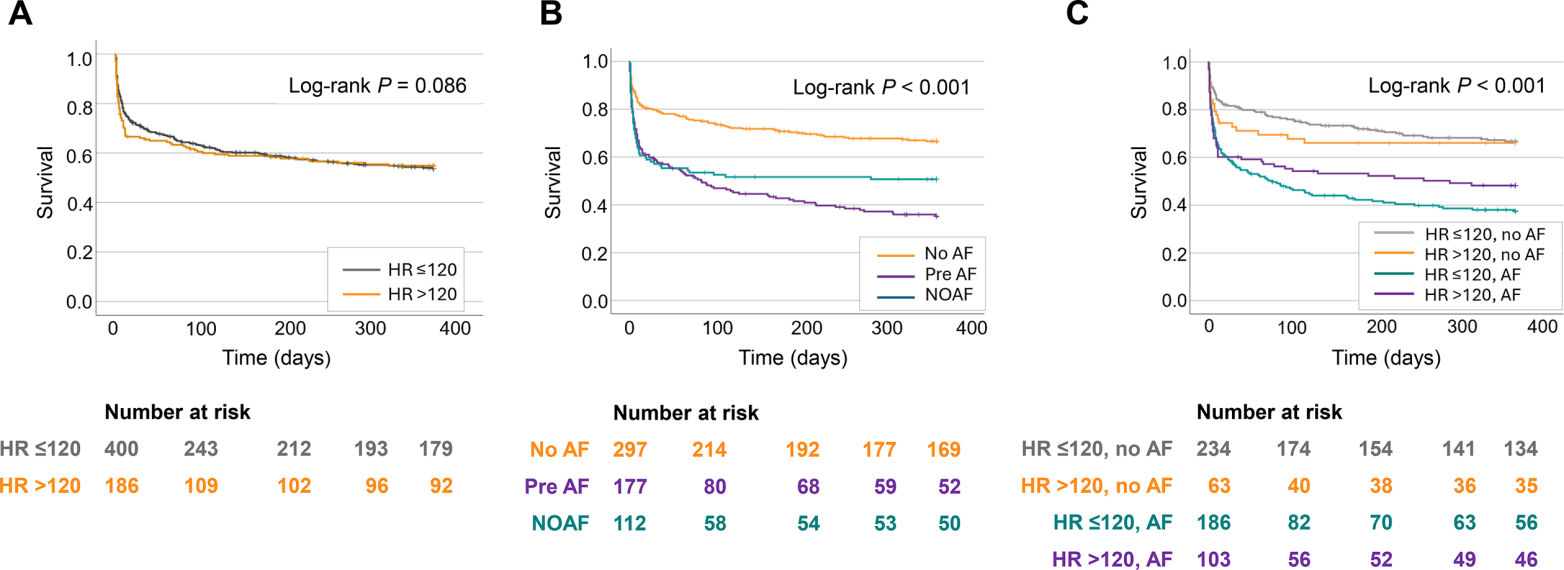
1-year mortality according to the highest recorded heart rate and atrial fibrillation status. Kaplan-Meier survival curves comparing patients (**A**) with the highest recorded heart rates ≤120 beats per minute versus >120 beats per minute (bpm), (**B**) according to AF group (no AF, pre-existing AF and new-onset AF) and (**C**) according to combined AF and heart rate categories. AF, atrial fibrillation; HR, heart rate; NOAF, new-onset atrial fibrillation; Pre AF, pre-existing atrial fibrillation.

At 1 year, the highest recorded heart rate was not associated with mortality, whether analysed as a continuous variable (HR 1.0; 95% CI 0.99 to 1.004; p=0.580), modelled using natural cubic splines (p=0.303) or dichotomised at 120 beats per minute (figure 2A). Compared with patients without AF, both pre-existing AF (HR 2.4; 95% CI 1.8 to 3.2; p<0.001) and NOAF (HR 1.8; 95% CI 1.3 to 2.5; p<0.001) were associated with higher 1-year mortality (figure 2B).

In stratified analyses of AF and heart rate, Kaplan-Meier curves demonstrated marked differences in unadjusted mortality between groups (figure 2C). Patients with a heart rate ≤120 beats per minute and no AF had the lowest mortality, followed by those with a heart rate >120 beats per minute and no AF. Mortality was higher in those with a heart rate >120 beats per minute and AF, and highest in those with a heart rate ≤120 beats per minute and AF.

These findings were consistent with univariable Cox regression, using patients with heart rate ≤120 beats per minute and no AF as the reference group (with the lowest mortality). Mortality was higher in those with heart rate >120 beats per minute and no AF (HR 1.1; 95% CI 0.7 to 1.8; p=0.652), those with heart rate >120 beats per minute and AF (HR 1.9; 95% CI 1.4 to 2.7; p<0.001) and those with heart rate ≤120 beats per minute and AF (HR 2.4; 95% CI 1.8 to 3.2; p=0.003).

**Figure 3:**
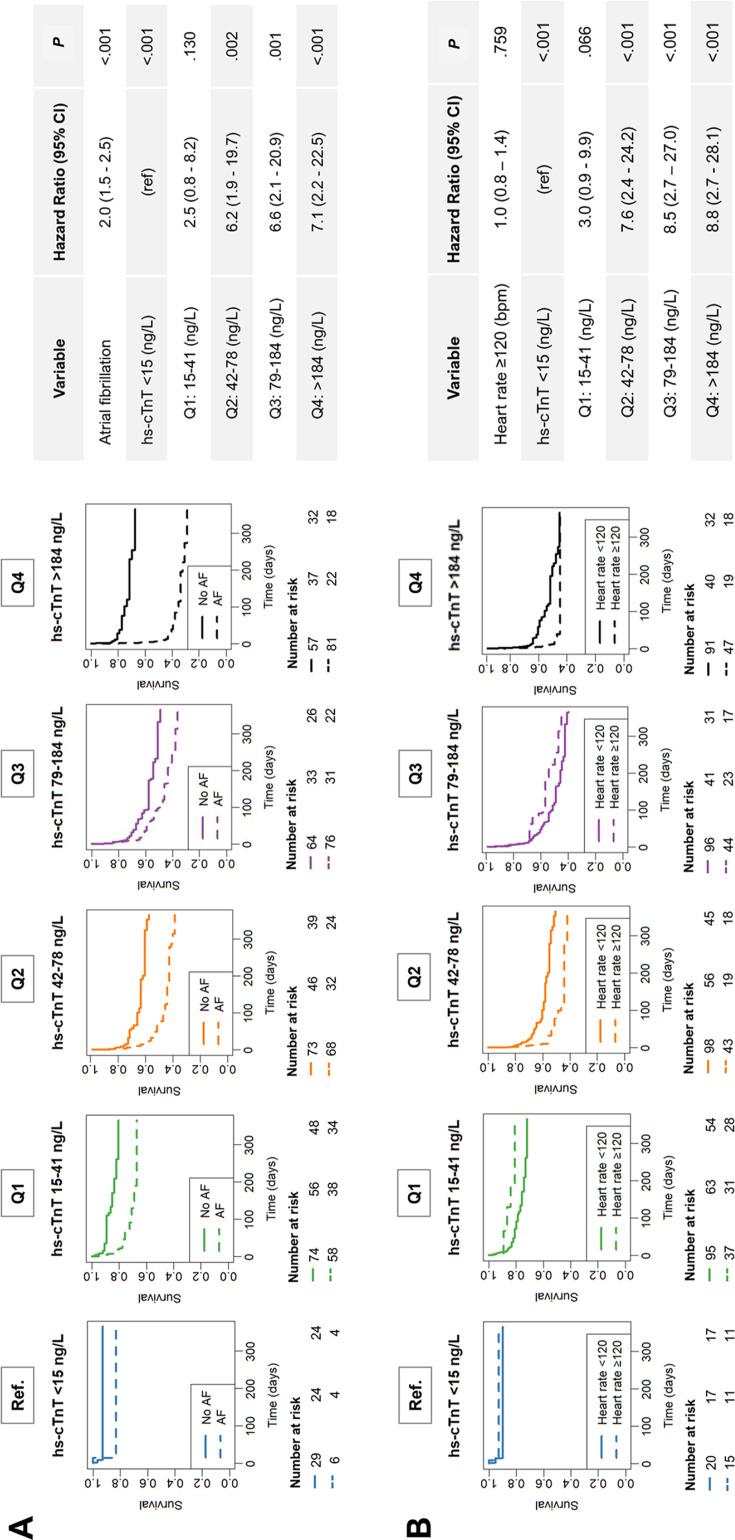
Association of high-sensitivity cardiac troponin T (hs-cTnT) concentrations with 1-year mortality by atrial fibrillation and heart rate status. Kaplan-Meier survival curves showing 1-year mortality in patients with sepsis stratified by (**A**) atrial fibrillation (AF vs no AF) and (**B**) highest recorded heart rate (≤120 beats per minute vs >120 beats per minute), across quartiles of highest hs-cTnT concentrations. Tables to the right display univariate Cox proportional hazards models for 1-year mortality, using hs-cTnT <15 ng/L as the reference category. AF, atrial fibrillation.

1-year mortality increased progressively across hs-cTnT quartiles, with an additive effect of AF (figure 3A). In contrast, mortality did not differ meaningfully by heart rate across hs-cTnT strata (figure 3B).

### Multivariable associations with mortality

In the multivariable model including AF, highest recorded heart rate and hs-cTnT, overall AF status and higher troponin concentrations were independently associated with 1-year mortality, whereas heart rate was not (table 2). The model was adjusted for age, sex, frailty, comorbid cardiovascular disease, laboratory markers reflecting renal function (creatinine), perfusion (lactate) and systemic inflammation (C reactive protein), as well as LVEF. Although LVEF was associated with higher 1-year mortality in univariable analysis (p=0.035), this association was no longer significant after multivariable adjustment.

**Table 2:** Multivariable associations with 1-year mortality

Variable	HR (95% CI)	P value
Atrial fibrillation		0.032
No atrial fibrillation	1.0 (reference)	
Pre-existing atrial fibrillation	1.4 (0.99 to 1.9)	0.055
New-onset atrial fibrillation	1.5 (1.1 to 2.2)	0.014
Log-transformed hs-cTnT (ng/L)	1.3 (1.1 to 1.4)	<0.001
Highest recorded heart rate (per 10 beats per minute increase)	1.0 (0.95 to 1.1)	0.992
Age (per year), years	1.02 (1.01 to 1.03)	0.003
Female sex	1.2 (0.9 to 1.5)	0.253
Clinical Frailty Scale (per point increase)	1.2 (1.1 to 1.4)	<0.001
Previous myocardial infarction	1.1 (0.7 to 1.5)	0.755
History of heart failure	1.4 (1.1 to 2.0)	0.019
Hypertension	0.9 (0.6 to 1.1)	0.277
Creatinine (per 10 µmol/L increase)	1.01 (1.001 to 1.02)	0.014
Lactate (per mmol/L increase)	1.13 (1.1 to 1.2)	<0.001
C reactive protein (per 50 mg/L increase)	0.91 (0.86 to 0.97)	0.002
Left ventricular ejection fraction		0.204
Echocardiography not performed	1.1 (0.8 to 1.5)	0.535
≥50%	1.0 (reference)	
40–49%	0.8 (0.4 to 1.3)	0.321
<40%	0.7 (0.5 to 1.1)	0.168

hs-cTnT, high-sensitivity cardiac troponin T.

## Discussion

### Principal findings

In this large cohort of critically ill patients with sepsis requiring vasopressor support, higher hs-cTnT concentrations and overall AF status were independently associated with increased 1-year mortality, whereas the highest recorded heart rate was not. Mortality increased progressively across hs-cTnT quartiles, with consistently higher risk in the presence of AF, suggesting an additive association. In secondary analyses, AF status explained only 1% of the variability in hs-cTnT concentrations, whereas heart rate explained none.

### AF, heart rate and troponin in the context of sepsis pathophysiology

Our findings should be interpreted within the broader clinical triad of AF, heart rate and troponin elevation, which commonly occurs during heightened circulatory and inflammatory stress in sepsis.^13^ These abnormalities likely arise from overlapping mechanisms, including adrenergic activation, dysregulated inflammation, intravascular volume shifts, transient myocardial strain, metabolic acidosis, fever and impaired autonomic regulation.^14 15^

The dissociation between heart rate and troponin concentrations observed in our study suggests that sepsis-associated myocardial injury may involve mechanisms beyond rate-related demand ischaemia alone. Similarly, the limited contribution of AF status to troponin variability argues against AF itself being the primary driver of myocardial injury in most patients. Instead, AF and troponin elevation may represent complementary markers of cardiovascular vulnerability and systemic disease severity during sepsis.

### Heart rate as a prognostic marker in sepsis

Evidence linking heart rate to outcomes in sepsis remains limited. Prior work in a non-sepsis AF cohort demonstrated a non-linear association between heart rate at emergency department presentation and hs-cTnT, with concentrations rising at ventricular rates ≥125 beats per minute.^8^ In sepsis, findings have been inconsistent: sustained tachycardia has been linked to higher short-term mortality in septic shock, whereas another study reported prognostic effects varying by lactate level, with faster rates associated with lower short-term mortality among patients with markedly elevated lactate.^7 16^ In a large sepsis cohort with AF, no difference in 30-day mortality was observed between patients with ventricular rates above versus below 120 beats per minute at intensive care unit admission.^11^

In our cohort, the highest recorded heart rate within the first 72 hours of sepsis onset was not independently associated with troponin concentrations or long-term mortality. One explanation for heterogeneous findings across studies is that heart rate in sepsis reflects multiple transient stimuli (eg, fever, hypovolemia, pain and adrenergic activation) alongside inflammatory or vascular processes with longer-term consequences.^13^ This complexity renders heart rate a fluctuating and physiologically non-specific marker, potentially limiting its independent prognostic value relative to AF (electrophysiological vulnerability) or troponin elevation (myocardial injury).

### AF as a marker of chronic vulnerability and acute physiologic stress

In contrast to heart rate, which reflects a dynamic physiological response, AF likely reflects underlying arrhythmogenic and cardiovascular vulnerability. Patients with pre-existing AF and those who develop NOAF during sepsis may represent a shared risk continuum characterised by comorbidities such as hypertension, heart failure, coronary artery disease, obesity and diabetes, conditions associated with chronic inflammation and atrial remodelling.^15 17 18^ During sepsis, acute inflammatory and haemodynamic stress may precipitate AF in predisposed individuals.^15^ Consistent with this concept, risk factors for NOAF during sepsis largely reflect acute illness severity, supporting the hypothesis that AF arises through the interaction between chronic cardiovascular substrate and acute systemic stress.^19^

### Interpretation of the paradoxical heart rate finding in AF

In patients with AF, the finding that those with heart rates ≤120 beats per minute had higher mortality than those with higher rates appears less paradoxical. A lower heart rate during sepsis may reflect conduction disease, structural heart disease or impaired autonomic reserve rather than effective rate control.^20^ An attenuated heart rate response to physiological stress has been associated with reduced haemodynamic reserve and adverse outcomes in other clinical populations.^21 22^ Accordingly, these findings are more consistent with underlying cardiac dysfunction than with higher heart rates reflecting a compensatory physiological response.

### Long-term outcomes and inflammation-resolution biology

When examining longer-term outcomes, for which prior evidence remains limited, our findings align with analyses by Walkey *et al.*^12 23^ Long-term vulnerability after sepsis may be understood within the inflammation-resolution framework.^15 24^ This model proposes that failure to transition from acute inflammation to specialised pro-resolving pathways results in persistent inflammatory signalling, fibrosis and tissue dysfunction in myocardial and atrial tissue over months to years.

Patients with AF, whether pre-existing or new-onset during sepsis, exhibit biological features that may increase susceptibility to incomplete resolution of inflammation. Pre-existing AF is characterised by impaired inflammatory homeostasis, atrial remodelling and fibrosis, whereas NOAF reflects heightened inflammatory and adrenergic stress that may trigger rapid atrial remodelling.^25 26^ Together, these processes promote an arrhythmogenic, pro-fibrotic substrate that impairs normal resolution of atrial inflammation after sepsis.^15^ Such post-inflammatory effects, including fibrosis, tissue degeneration, impaired function and arrhythmogenic substrate formation, may help explain the independent association between AF and 1-year mortality observed in our cohort.^15^

### Troponin, AF and long-term cardiovascular risk

The combined prognostic significance of troponin elevation and AF in sepsis has received limited investigation, with most analyses treating troponin as a marker of severity and AF as a comorbidity.^3 27^ In broader AF populations, however, high-sensitivity troponin independently predicts cardiovascular events and mortality, suggesting that troponin reflects clinically meaningful myocardial injury rather than transient physiological stress alone.^28^

AF frequently coexists with heart failure and ischaemic heart disease in a bidirectional relationship that promotes structural and functional decline.^10 26^ Sepsis survivors are themselves at increased risk of subsequent cardiovascular events, and infection-related AF frequently recurs after discharge.^12 29^ Within this broader context of cardiovascular vulnerability, the coexistence of elevated troponin and AF in our cohort identified a subgroup at increased risk of long-term mortality.

### Clinical significance

Plasma troponin elevation in sepsis should not be dismissed as a consequence of AF or higher heart rate, as mechanisms of myocardial injury likely extend beyond rate-related demand ischaemia. The coexistence of troponin elevation and AF identifies a subgroup of sepsis survivors at increased risk of long-term mortality. These findings highlight the need for further research to clarify the mechanisms underlying sepsis-induced myocardial injury and to determine whether structured postdischarge follow-up, cardiovascular assessment or targeted risk-reduction strategies improve outcomes.^30^

### Strengths and limitations

This study has limitations. Its observational design makes it vulnerable to bias and precludes causal inference. As a single-centre study, the generalisability of findings to other sepsis populations and care settings may be limited. Plasma hs-cTnT testing was not protocolised but performed at clinicians’ discretion, introducing potential selection bias towards patients with suspected cardiac disease, electrocardiographic abnormalities and haemodynamic instability.

Continuous rhythm monitoring occurred throughout the critical care stay and AF episodes were documented in the medical record; however, AF duration and burden were unavailable, preventing evaluation of arrhythmia burden or recurrence as contributors to long-term outcomes. Similarly, the use of the highest recorded heart rate within 72 hours may not have adequately captured cumulative tachycardic burden or myocardial stress during sepsis, potentially attenuating associations between heart rate, troponin release and mortality. In addition, echocardiographic data were only available in a subset of patients, which may have introduced residual confounding and limited complete adjustment for underlying cardiac dysfunction in multivariable analyses. Although multivariable adjustment included major cardiovascular comorbidities, frailty, renal dysfunction and illness severity, residual confounding from unmeasured or incompletely measured factors cannot be excluded.

The study also has important strengths. To our knowledge, this is the first study to jointly evaluate AF, heart rate and high-sensitivity troponin in relation to 1-year outcomes in sepsis. The use of a large, well-characterised EHR cohort and complete 1-year mortality follow-up strengthened the robustness of the findings.

## Conclusions

In adults with sepsis requiring vasopressor support, AF and elevated hs-cTnT were associated with increased 1-year mortality, with additive prognostic value independent of heart rate. Concomitant AF and troponin elevation identify a high-risk subgroup of sepsis survivors whose myocardial injury should not be attributed solely to rate-related demand ischaemia. Further research is needed to clarify underlying mechanisms and to determine whether targeted cardiovascular assessment or treatment strategies improve outcomes in this population.

## Supplementary material

10.1136/openhrt-2026-004073 online supplemental file 1

## Data Availability

Data are available upon reasonable request.
