# Supplementary material for: Rethinking rate-related myocardial injury in sepsis: atrial fibrillation, heart rate, cardiac troponin T and long-term mortality
Source: Open Heart. 2026 Jun 22;13(1):e004073. doi: 10.1136/openhrt-2026-004073 (PMC13289210; doi:10.1136/openhrt-2026-004073)
Supplement: online supplemental file 1 [file openhrt-13-1-s001.docx]

**Supplemental Material**

**Figure S1.** Flowchart of Patient Selection for the SET (Sepsis and Elevated Troponin) study.^3^


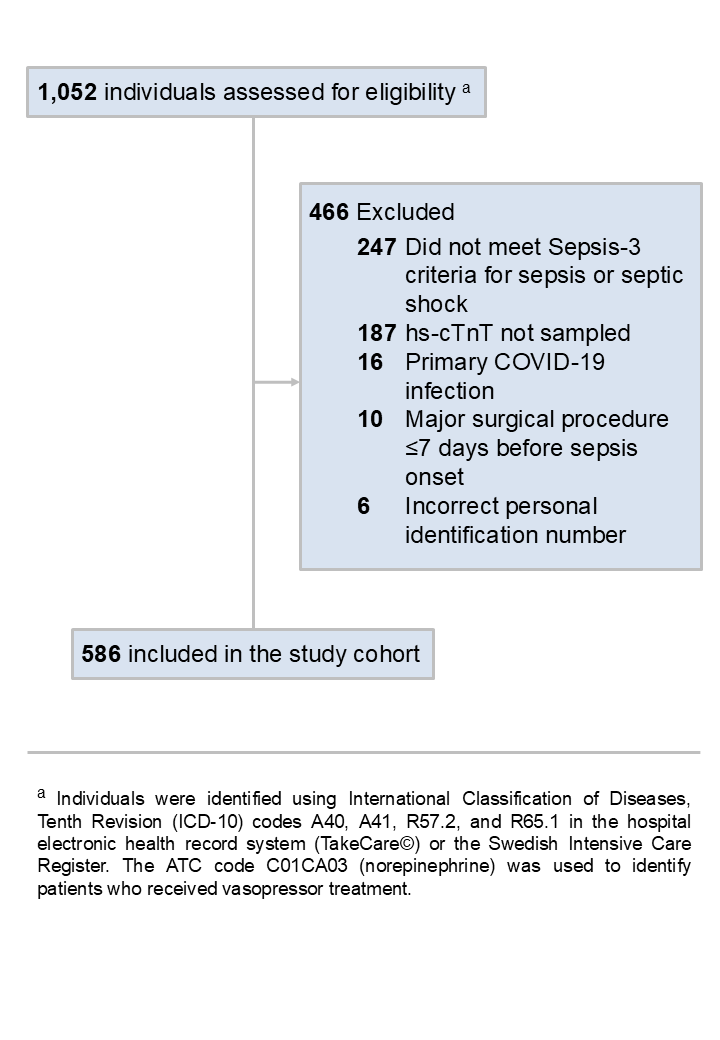


^a^ Individuals were identified using International Classification of Diseases, Tenth Revision (ICD-10) codes A40, A41, R57.2, and R65.1 in the hospital electronic health record system (TakeCare©) or the Swedish Intensive Care Register. The ATC code C01CA03 (norepinephrine) was used to identify patients who received vasopressor treatment.
